# Research on the Influence of AI and VR Technology for Students’ Concentration and Creativity

**DOI:** 10.3389/fpsyg.2022.767689

**Published:** 2022-03-24

**Authors:** Qiming Rong, Qiu Lian, Tianran Tang

**Affiliations:** ^1^School of Film and Television Animation, Guangdong Literary and Art Vocational College, Guangzhou, China; ^2^Guicheng Senior High School, Foshan, China; ^3^Artificial Intelligence College, Dongguan Polytechnic, Dongguan, China

**Keywords:** teaching mode, virtual reality, artificial intelligence, deep learning, creativity

## Abstract

The application of digital technology in teaching has triggered the evolution of traditional teaching. Students have different corresponding relationships under digital behavior. The interactive technology of artificial intelligence (AI) and virtual reality (VR) provides a new driving force for the development of art education and psychology. Firstly, this thesis analyzes the limitations and existing problems of traditional art education. Especially, the influence of the teaching mode of art education on the teaching of other disciplines develops a targeted student-centered digital education program. Secondly, the author used VR equipment and technology to let students experience the virtual world freely, and then, the relevant data model was established on the basis of analyzing the reasons affecting students’ creativity and concentration. Thirdly, the data model was applied to art education in order to improve students’ concentration and creativity. Then, the author compared and analyzed the data of the students under different teaching models through questionnaires. The results show that introducing VR and AI technology into art education and encouraging students to carry out deep learning can significantly improve student concentration and creativity. Finally, the influence reasons are analyzed from the perspective of psychology. VR interaction and Artificial Intelligence can be introduced into middle school fine art education which is to the benefit of students’ deep learning, thus students’ concentration and creativity can be improved.

## Introduction

Nowadays, people all pursue a better life; thus, art and artistic forms frequently appear in daily life. People’s ideas are also subtly changed by art, and more parents and schools attach importance to art education. Therefore, as an important form of art, the fine art is widely respected. The school is also improving the level of art education to meet the development requirements of the present society and cultivate students’ artistic and creative ability. VR technology breaks the limitation of traditional two-dimensional classroom and provides students with a more realistic and tridimensional visual space (Li, 2017).

Nowadays, the application research of AI and VR technology in the field of education is in full swing. The detailed parameters of graphics and two-dimensional and tridimensional color analysis can be detected by the relatively sophisticated AI technology, so that middle school students can more intuitively find the difference in terms of graphics, structure, light, and color. VR technology can elevate the sense of experience in fine art to a higher level, improve students’ interest in fine art learning, provide students with more perspectives of observing the world, and cultivate students’ core literacy in fine art. Therefore, the significance of AI and VR technology is obvious, which will play a key role in the future middle school fine art teaching (Zhao, 2019; He, 2020). The composition of teaching model is diverse. We can apply AI and VR technology as the teaching means, adopt deep learning model as the teaching method, and combine technology with teaching content to enrich presentational forms and enhance image memory for students. Let students form understanding memory in learning; therefore, they can apply the knowledge to practice after deep learning and use the knowledge flexibly. Adopting the hybrid evaluation mechanism can discover the student potential, improve their cognitive and interpretative ability of fine art, and stimulate their inner creativity. This is also a beneficial exploration of the application of digital technology in traditional teaching reform (Tang, 2019; Liu, 2020).

Fine art teaching has developed for many years, while the traditional model has to give space for the new media model in this information age. Art curriculum standards request teachers to use the Internet and new media in fine art teaching flexibly, enrich the fine art classroom, and broaden students’ thinking by using the Internet resources. Teachers should also encourage students to take the initiative to collect and sort out information, and use computers or digital cameras to create. As fine art courses are practical, teachers should guide students to use new media or traditional media to develop their imagination and creativity. Schools can cultivate students’ diversified esthetic concepts and humanistic qualities by fine art courses, thus realizing esthetic education (Shao, 2019).

## Literature Review

We have consulted many materials and articles related to teaching theory research, as well as the advantages and disadvantages of traditional education mode, multimedia education mode, and modern education mode summarized from our research and practice of daily teaching.

### Traditional Educational Mode

In the traditional model, the status of fine art is not as high as it is now. As a part of quality education, fine art education is often put at the end, so the standard is not high from art teachers to students, let alone the art appreciation and painting ability of students. In the traditional model, the way of teachers’ education is still in the period of essentialism education. The teacher-centered teaching concept seriously fetters students. Students participate in class passively in the single interaction model (Johnson-Glenberg, 2018; Zheng, 2020). Traditional cramming teaching method directly leads to the result of disconnection between teaching and learning of teachers and students, and there are very few interactions in class. Thus, a series of problems will appear: students have no interest in fine art; teachers teach very hard; students’ learning effect, initiative and creativity are not enough; the raise of students’ esthetic appreciation ability is not obvious; and what students have learned is easy to forget (Edwards et al., 2019; Maas et al., 2020; Sahin and Yilmaz, 2020). Unilateral indoctrination for students and only focusing on the knowledge will ultimately have little effect. In the long run, students will gradually lose interest in learning fine art and receive less and less knowledge (Yilmaz and Goktas, 2017; Huang et al., 2019a; Özgen et al., 2021).

### Multimedia Educational Mode

The addition of new media model is the innovation of education industry. Teaching resources become rich increasingly, especially there are extensive input of modern art. So, people can use the media to show more intuitive structure and lighting effects, and the beauty of Chinese ink painting and other fine art works can also be showed, which is advantageous for the contrast between Chinese and western art and painting skills (Chen et al., 2015, 2020a, 2021). The single traditional teaching model has also been changed to the interactive teaching under the new media model, which has the characteristics of intuitive image and timely feedback. Reasonable use of new media technology creates conditions for the realization of interactive fine art teaching. Teaching work is the combination of “teaching” and “learning,” so we can neither ignore the teaching work of teachers nor the learning situation of students. Student-centered teaching means that teachers need to understand the knowledge reserve of students, collect, and sort out the information of students. Teachers flexibly design teaching links in class and creates interactive teaching situations, so that the students become more active. In this way, we can exercise students’ brains, train students to think from different perspectives, learn a variety of artistic expressions, and improve students’ ability to use information technology and media literacy. Students participate in fine art activities in the way of individual or collective cooperation, experience the happiness of learning fine art, and improve competence to communicate and cooperate. However, under the new media model, the “mouse party” has also emerged. The interactive form of interactive courseware is single, which is operated by mouse click. This single approach has two drawbacks. On the one hand, the computer and other multimedia auxiliary teaching courseware have become the slide show, its interaction has not been greatly developed. For example, in some teaching software and web pages, students can click the button to check the relevant content, and the computer will push some learning content according to students’ preferences (Huang et al., 2019b; Chen et al., 2019b, 2020). However, these teaching contents are presented in the form of images, and interpersonal interaction is only reflected in the action of clicking the mouse. Although the teaching effect is different from the traditional teaching model, its essence does not change much. On the other hand, a single form of interaction can make students bored. When students first contact interactive courseware, they will take the initiative to learn because of the freshness. However, after using the mouse for a long time, they will inevitably become numb. The freshness will gradually disappear, and students will gradually lose interest, especially middle school students who is in the period of having a strong exploratory psychology for all things. Thus, the teaching effect is bound to be greatly reduced (Liu et al., 2014; Huang et al., 2015, 2017; Lin et al., 2020).

### Modern Educational Model

With the mature and promotion of AI and VR technology, many students will be exposed to VR technology in daily life, travel, and games. In the help of eyes and wearable devices such as helmets, VR technology can put people into the scene, so that people are immersed in it and interact with the landscape. In this way, students have inquiry-based learning under the guidance of interest, take the initiative to acquire knowledge, and feel the artistic beauty of the art of painting. In VR interactive learning, students can also be guided by questions which means teachers set tasks for them and give answers during the experience to arouse their desire for knowledge (Ye et al., 2019, 2021; Fu et al., 2020). In the course of teaching, teachers can slip in answers to eliminate students’ doubts, guide students to actively participate in the class, carry out deep learning, and improve the efficiency of the class. The teaching behavior that teachers respond to students’ one by one, which is not only conducive to the internalization of students’ knowledge in a harmonious and equal relationship, but also meets the psychological characteristics of middle school students and arouses their learning initiative. Students realize that teachers do not teach fixed courseware at a high level but participate in students’ active learning. In this good model and atmosphere, students are more willing to learn deeply, internalize knowledge, keep learning new and interesting things, and cultivate their own concentration and innovation ability.

In general, the direction of education is to improve students’ core literacy in fine art, esthetic and discrimination ability, and be able to live and communicate with an appreciative sense, and ultimately affect students’ concentration and creativity.

## Research Model and Framework

### Research Model

**Figure 1:**
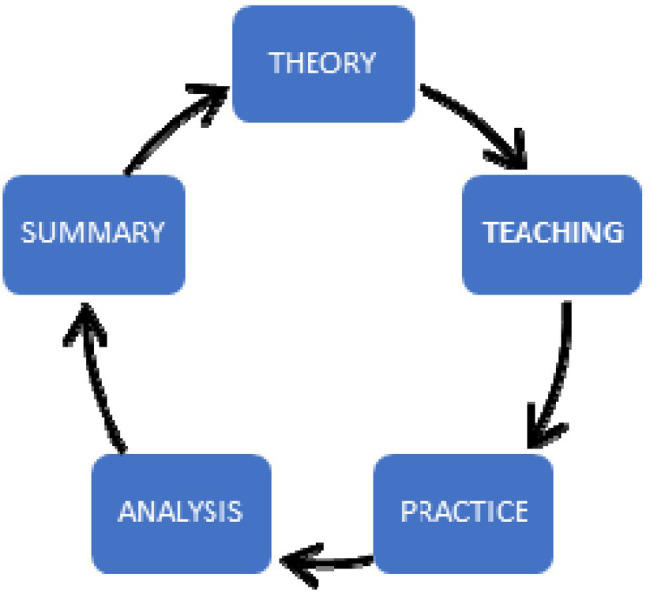
Theoretical of research methods.

**Figure 2:**
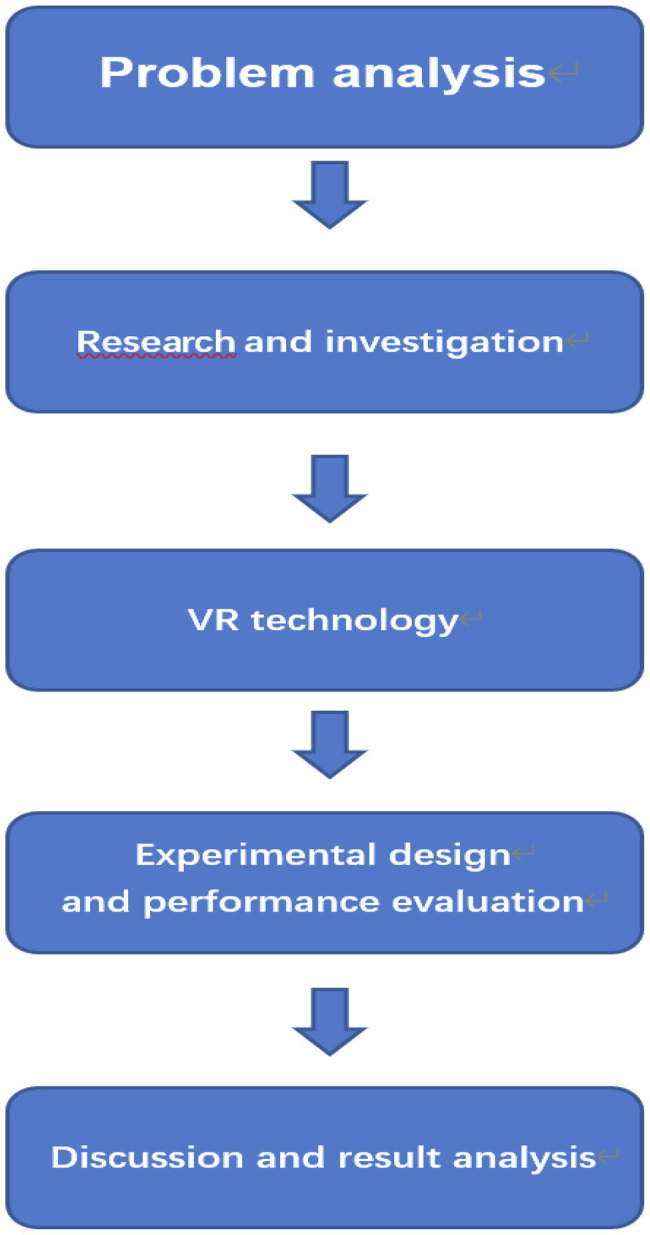
The research process of proposed flowchart.

Theory guides the development of practical training as Figure 1 theoretical of research methods. Practice verifies the feasibility of the theory. The main research process as Figure 2 is investigation and experiment. Investigate the basic situation of students and teachers, and select appropriate samples for the experiment. Deep learning teaching design of middle school students under the guidance of AI and VR technology. We adopt chart research and operation methods for operation (Li, 2017).

#### Reinforcement Learning Theory

From the perspective of psychology, Skinner believed that all behaviors are composed of reflexes, and people strengthened the depth of learning in the process of continuous stimulation and feedback.^[11]^ He believed that people could acquire knowledge and ability under constant stimulation by the repeating practice of mice press down pedaling. He also introduced operational learning into the classroom, emphasized behavioral goals and feedback in the classroom, and created the famous program teaching and teaching machine, which had a great impact on teaching (Li, 2017).

#### Multiple Intelligences Theory of Howard Gardner

The multiple intelligences theory proposed by Gardner is a widely recognized theory in modern education field. It breaks the view of single intelligence of human beings and believes that human intelligence can be divided into eight kinds, that is to say, people can think and learn from multiple dimensions and levels. AI and VR technology are more multi-dimensional learning in perception, interaction, vision, language, and movement. Visual–spatial intelligence refers to the ability to feel, distinguish, remember, and change the spatial relationship of objects, and to express thoughts and feelings through it. It is shown as the sensitivity to lines, shapes, structures, colors, and spatial relationships, as well as the ability to express them through plane graphics and tridimensional modeling.^[12]^ Multiple intelligences theory holds that every child has the opportunity to become a talent, which can provide reference for teachers’ evaluation in teaching and arouse students’ stronger desire to explore fine art. Through observation and learning, students can exercise thinking, broaden the horizon and increase esthetic ability. Teachers also improve their teaching methods and models in the interaction, so as to evaluate students’ abilities and works more scientifically and give them more positive guidance (Li, 2017).

#### Constructive Learning Theory

Piaget, the Swiss psychologist who proposed constructive learning theory, believed that knowledge learning actually had two processes. The first is that students assimilate the external knowledge into their own knowledge. The second is that when the external environment changes, students should adapt to the external changes and reconstruct the existing knowledge. These two processes: one is to expand students’ knowledge accumulation; the other is to change the existing knowledge structure according to the changes of the outside world.^[14]^ Therefore, in the process of learning, students should pay attention to the knowledge they are learning, and the accumulation of knowledge in the past is also important. While learning new knowledge, students should analyze and compare it with the previously learned knowledge, identify their rationality, the similarities and differences, process and refine the knowledge freely, and finally complete the learning process.

#### Deep Learning Theory

The concept of deep learning is based on the research of brain science. Through the research of human brain’s deep learning, a new door of machine learning has been opened in the field of science and technology, in which the machine completes autonomous learning and analysis by simulating the thinking process of the human brain and finally responds to instructions. First of all, deep learning is a kind of ability based on understanding (Li, 2017). Only one has its own critical thinking about the content and knowledge learned, and connects with previous knowledge points, transfers and transforms new and old situations, can he make decisions and solve problems. Secondly, deep learning focuses more on the integration of information, reconstruction of the knowledge system through its own thinking, and proactively solving existing problems. Through deep learning art courses, students can judge the talents that should be transferred according to different situations, have a clearer judgment on problems, and can solve related problems encountered, even associative problems. Lastly, shallow learning and deep learning are not in conflict, which is a single continuous whole body. When learning new knowledge, students are required to critically understand and learn, and find out the interrelated parts of the new and old knowledge, which may be the learning idea of “Longmen Grottoes” art appreciation, or the connection between “Louvre” and “Pyramid,” and find the same items in art to merge and sort out the dissimilarity.

### Research Method

#### Set Goals

The first is to determine the teaching subject. With the development of new media, online museums, art exhibitions, cloud classes, and other modes have emerged one after another, making people think about which form is the most attractive. There are also many teaching models, such as interactive learning, deep learning, inquiry learning, and blended learning, appearing in different schools, and facing students of different ages. Some teachers directly copied these models but ended up with little effect. Is this a problem of educational philosophy? Of course not, because students in different regions and at different ages have different acceptance. Therefore, in the initial stage of curriculum design, it is necessary to locate the age of students and teaching goals, so as to guide the overall difficulty of the class. Only by setting up a standard in advance can we know how far the students are from the standard and adjust the course in time. Secondly, teachers should determine the goals of AI and VR technology in use, that is, which key and difficult problems in fine art teaching need to be solved through the use of AI and VR equipment. Thirdly, it is necessary to ensure that the combination of AI and VR technology can be used in teaching practice, so as to improve class efficiency in a limited time. Finally, teachers should set goals for students in classroom interaction, let students know what to do and understand, so as to gain a sense of learning experience and accomplishment.

#### Focusing on the Classroom Experience

**Figure 3:**
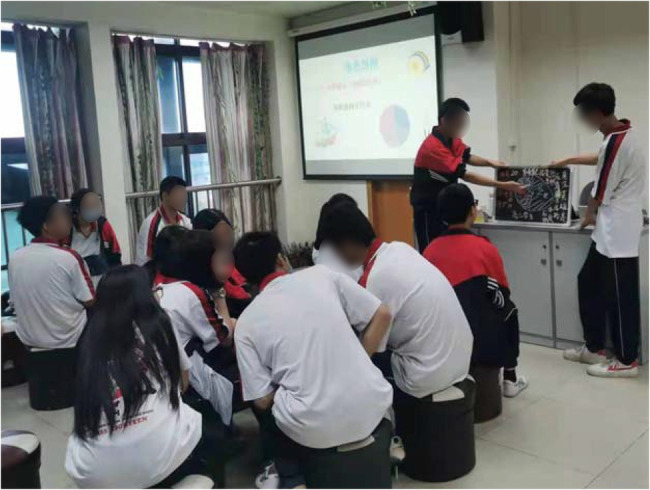
The students participate discussion in the experiment.

**Figure 4:**
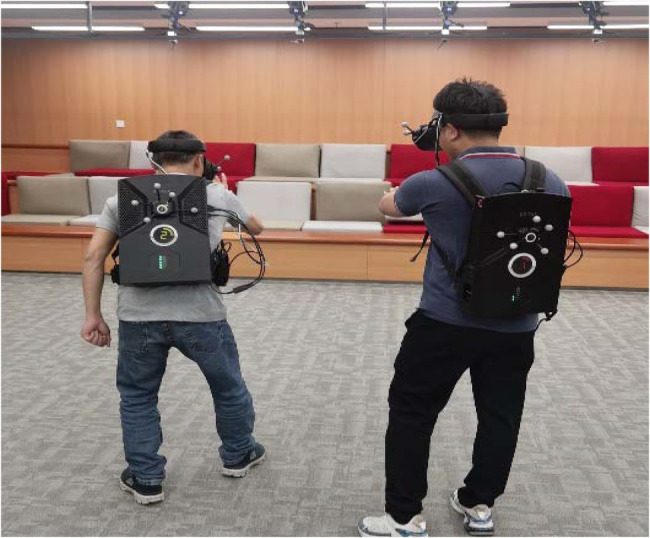
Photos of students participating in the experiment.

The student is the subject of the classroom, the teacher is the guide and the answerer, and the equipment is the tool as Figure 3. The students participate discussion in the experiment. To clarify the relationship between the three, students can be divided into several groups, and answer the questions encountered in the course in a unified way as Figure 4.

Deep learning under AI technology can be multifaceted, for example, it can be the appreciation and reference of the artistic style of western art masters, or the color module and group analysis. After the AI program is set up, the students can propose the direction of exploration and give the initial impression, and then obtain a combined analysis of the color blocks through the process of AI technology. Works such as Van Gogh’s sunflower, it can first be determined as warm tone. Then, students will be asked to discover the relationship between cold and warm tones, light and dark in the work through observation, then use AI technology to clearly mark the relevant position, and compare the differences between human observation and AI technology analysis. The color three-dimensional model in VR can also be used to analyze the combination law between the approximate colors of a certain color in the image, so as to cultivate students’ observation and color matching ability. What is more, the novelty of VR equipment allows children to discover, solve, and explore the colors and beauty. “The source of human touch is our senses, including the eyes, ears, nose, tongue, body and tactus. If we close our senses, we will kill the roots of touch. Of course, we will not see its buds and fruit.” The experience of being touched is brought about by our human body sensory system. There are five types, commonly known as the five senses, which are vision, hearing, taste, touch, and feeling. Traditional projected art works focus on visual and auditory feelings. According to the analysis data of relevant researchers, visual perception occupies the most important position in the sensory system, because the visual image created by a series of text, pictures, videos, and other visual languages can convey effective information and emotions.^[17]^ In terms of specific connotation, deep learning focuses on the transfer and application of knowledge, which not only requires students to have a high degree of ability to understand the content but also requires students to have a deep understanding of the learning situation. Only by grasping the key elements of the situation can we clarify the difference, and make accurate and clear judgments on the new situation, so as to realize the transfer and application of the principles and methods. At last, teachers should give supplementary explanations on some problems. This can arouse the enthusiasm of middle school students, let them discover the truth in practice, and finally let the students end the course in the exercise of concentration and curiosity.

#### Feedback After Class

Whether it is education or enterprise, customer experience is an important direction for product improvement. A complete evaluation and effective feedback are an important way to realize the self-recognition and adjustment of students’ learning status and deep learning, which can improve students’ understanding of knowledge and learning methods, assist teachers to adjust teaching methods in time, and promote the effectiveness of classroom teaching. Deep learning also requires teachers to focus on the development of students’ metacognitive abilities and thinking qualities in the evaluation process, because the developed metacognitive abilities and improved thinking qualities deeply stimulate learning motivation, and only then will students be introduced to a higher level.

## Experimental Design and Performance Evaluation

The research object and method on the concentration and creativity of middle school students.

### Experimental Design and Time Arrangement

In this research, a controlled experiment was used. The experimental group received AI and VR fine art training, and the control group received traditional training. In order to ensure the effectiveness, the experimental group used two nights and one afternoon every week to conduct AI and VR training courses led by art teachers for 20 min each time. In the rest time, students left the equipment for free hand-painting creation training, that is, the weekly AI and VR course training includes 3 group training and 4 free hand-painting creation training, and after the training is completed, the training evaluation form is filled out, which lasts for four consecutive weeks.

### Questionnaire

In the first year of high school students, 31 students (11 boys and 20 girls) with a score greater than or equal to 50 in the “Creative Thinking Test for Middle School Students” were selected to participate in the experiment, and 17 of them (8 boys and 9 girls) participated in the experiment group and filled in “Participating in the experimental agreement.” All training is completed, and the effective data recovery rate is 100%. Questionnaire include: 1. Why choose XR Development with Unity. 2. What you receive. 3. What you learn in the course. 4. How the course is taught. 5. How to become an AR/VR Developer.

### Research Tool

The AI and VR course training video contains four themes: color mixing, graphics, mechanism, and landscape photos. “Creative Thinking Test for Middle School Students,” “Distraction Scale,” and “Test Anxiety Scale.” The above three questionnaires were collectively tested to students before and after the AI and VR course training. Critical Reasoning Ability Test Suite: Logiks is a Critical Reasoning Ability Test suite built by international psychometric testing publisher, PSI and made available to you *via* countests. Critical Thinking ability is consistently ranked as an essential criterion for success in management and professional roles by employers and accounting professional bodies alike.

Aimed at people who have completed tertiary level education and for professional, management, and graduate roles, the Logiks Critical Reasoning test includes as: Critical Verbal to test how effective a candidate is in comprehending complex written text, separating true from false information, strong from weak arguments, and drawing logical conclusions. Critical Numerical Reasoning to test effectiveness in turning data into meaningful and accurate management information by spotting trends and outliers and making fast and accurate calculations. Reasoning to test how quickly your candidate can grasp concepts outside of their previous experience, comprehend abstract patterns such as workflows, and whether the time, effort, and money invested in their training is likely to yield results.

### Statistical Method

Using SPSS 22 statistical software to perform paired T test on the effective data of the experimental group. The IBM^®^ SPSS^®^ software platform offers advanced statistical analysis, a vast library of machine learning algorithms, text analysis, open source extensibility, integration with big data, and seamless deployment into applications.

Its ease of use, flexibility, and scalability make SPSS accessible to users of all skill levels. What is more, it suitable for projects of all sizes and levels of complexity, and can help you and your organization find new opportunities, improve efficiency, and minimize risk. Within the SPSS software family of products, SPSS Statistics support a top-down, hypothesis testing approach to your data while SPSS Modeler exposes patterns and models hidden in data through a bottom-up, hypothesis generation approach.

### Result

Changes in anxiety levels before and after training in AI and VR courses. After AI and VR course training, students’ test anxiety and distraction levels were significantly reduced (*p* < 0.05), while creativity was obviously improved (*p* < 0.05), as shown in Table 1.

**Table 1:** Paired *T*-test results of students’ creativity, distraction, and anxiety levels before and after AI and VR course training.

	Mean	SD	F
Distraction (before)	111	13.88794	0.014
Distraction (after)	95.4118	22.40552	
Creativity (before)	125.2353	19.56569	0.001
Creativity (after)	133.824	14.3057	
Anxiety levels (before)	56.3529	11.59710	0. 002
Anxiety levels (after)	45.7647	17.35846	

Applying AI and VR technology to the fine art course training of middle school students can effectively improve students’ creativity and concentration, and at the same time, reduce students’ test anxiety. On the whole, middle school students are relatively efficient and active in class, which can well stimulate their enthusiasm for learning and creativity, especially about students’ love of fine art. After long-term deep learning, the knowledge of art and the way of thinking will be greatly improved.

### Discussion and Exploring the Reasons of Influence Based on Psychological Theories

German sociologist Jurgen Habermas believed that any communicative behavior required authenticity, sincerity, and effectiveness. The communicative behavior is accomplished in dialogue through language as a medium, thereby forming the behavior of mutual understanding and communication between the interlocutors. The realization of communication behaviors needs to meet these conditions: under the common social norms, the recognition and comprehension of these norms should be achieved in both parties; the appropriate text, symbols, and other media should be used within the scope of others’ understanding; and the communication under an equal status creates a harmonious and free communication atmosphere. From a psychological point of view, it is to return the classroom to the students to create a sound and free learning environment for the students. In this atmosphere, the communicative behavior is more easily recognized and accepted. Teaching is a mutual process, that is, output–input knowledge through the teaching model and the student’s autonomous learning model. In the initial stage of fine art recognition, such positive interaction is relatively unrealistic, that is, the deep learning does not conflict with shallow learning mentioned above. For shallow learning, if there are equipment similar to AI and VR and the guidance of teachers, it is very likely that students will start to learn actively and turn their questions into motivation to find answers, thus achieving the effect of deep learning. According to Vygotsky’s “Zone of Proximal Development” theory, the purpose of this theory is to promote the cognitive development of adolescents, which refers to the individual who cannot complete the task independently but with the help of teacher or peers, that means the transitional stage between the current level of the youth and the level to be reached. There are many subjects in the middle school stage, and the teaching evaluation is mostly based on the performance of the students, which eventually leads to the gradual decline of students’ interest in subject learning and stagnant cognitive abilities. While the addition of VR technology will enrich the fine art teaching methods in middle schools, and AI technology will make the analysis of fine art teaching more intuitive. With the help of AI and VR technology, students whose physical and mental development is in the “Zone of Proximal Development” can increase their interest in fine art learning and stimulate learning potential. For example, teaching in virtual art museums can create simulation art museums and art works for students through VR technology, enhance students’ perceptual understanding during the learning process, and use new technologies to guide students in fine art learning. For middle school students, their cognition is at the peak. Compared with elementary school students, they have richer knowledge reserves, more concentration, and curiosity. Deep learning under AI and VR technology makes the classroom more interesting and enriching, and the explanation of knowledge points is more accurate and clearer. New technology-driven teaching, with the attraction of equipment and the guidance of teachers, enriches the concentration and creativity of students, which is also reflected in other subjects. The curiosity of middle school students on things is also the reason why AI and VR technology can be successfully introduced into the classroom. In terms of behavioral influence, middle school students’ imitation and social skills have also improved, and blind obedience in learning is also the reason for the success of deep learning in fine art classroom.

## Conclusion

The introduction of AI and VR technology into the campus is a reform of the curriculum model, and the advantage of VR/AI technology is not only to create a virtual teaching scene for students but also to enhance the interaction and communication between various classroom elements.

This work effectively and unequivocally contributes to the improvement of teaching, namely, teaching through the use of artificial intelligence; on the other hand, it highlights the new role of students and the advantage they reveal in the learning path, but also in the attitude of entrepreneurship, with the use of artificial intelligence equipment. From a psychological point of view, the interaction of human intelligence with artificial intelligence makes an excellent contribution here as it exemplifies that in a school this same interaction increases students’ attention and creativity. Of course, our experiment also has certain limitations, which cannot fully reflect the impact of AI and VR technology on teaching effect. We just made a beneficial exploration in the direction of teaching reform. Finally, it is also a contribution to the teaching of art and to the development of the psychology it addresses, as well as to art itself. The great contribution lies in the triggering of the transition between traditional teaching and digitalized techno-behavioral teaching. The approximations between cognition and digitization are not always concordant and much less exemplary of receptivity as long as everything refers to knowledge, above all in the area of psychology. It is a very precise but very subjective agenda and it deserves the most attention due to being the object of speculation.

## Data Availability Statement

The original contributions presented in the study are included in the article/supplementary material, further inquiries can be directed to the corresponding author.

## Author Contributions

All authors contributed to the study conception and design, Material preparation, data collection, and analysis performed by QL and TT. The first draft of the manuscript was written by QR. All authors contributed to the article and approved the submitted version.

## Conflict of Interest

The authors declare that the research was conducted in the absence of any commercial or financial relationships that could be construed as a potential conflict of interest.

## Publisher’s Note

All claims expressed in this article are solely those of the authors and do not necessarily represent those of their affiliated organizations, or those of the publisher, the editors and the reviewers. Any product that may be evaluated in this article, or claim that may be made by its manufacturer, is not guaranteed or endorsed by the publisher.

## Appendix

Questionnaire includes as:

1. Why choose XR Development with Unity.
2. What you receive.
3. What you learn in the course.
4. How the course is taught.
5. How to become an AR/VR Developer.
